# Role of Microbiota and Innate Immunity in Recurrent *Clostridium difficile* Infection

**DOI:** 10.1155/2014/462740

**Published:** 2014-06-05

**Authors:** Stefano Bibbò, Loris Riccardo Lopetuso, Gianluca Ianiro, Teresa Di Rienzo, Antonio Gasbarrini, Giovanni Cammarota

**Affiliations:** A. Gemelli Hospital, Division of Internal Medicine and Gastroenterology, Department of Internal Medicine, School of Medicine and Surgery, Catholic University, 8, 00168 Rome, Italy

## Abstract

Recurrent *Clostridium difficile* infection represents a burdensome clinical issue whose epidemiology is increasing worldwide. The pathogenesis is not yet completely known. Recent observations suggest that the alteration of the intestinal microbiota and impaired innate immunity may play a leading role in the development of recurrent infection. Various factors can cause dysbiosis. The causes most involved in the process are antibiotics, NSAIDs, acid suppressing therapies, and age. Gut microbiota impairment can favor *Clostridium difficile* infection through several mechanisms, such as the alteration of fermentative metabolism (especially SCFAs), the alteration of bile acid metabolism, and the imbalance of antimicrobial substances production. These factors alter the intestinal homeostasis promoting the development of an ecological niche for *Clostridium difficile* and of the modulation of immune response. Moreover, the intestinal dysbiosis can promote a proinflammatory environment, whereas *Clostridium difficile* itself modulates the innate immunity through both toxin-dependent and toxin-independent mechanisms. In this narrative review, we discuss how the intestinal microbiota modifications and the modulation of innate immune response can lead to and exacerbate *Clostridium difficile* infection.

## 1. Introduction

Bacteria residing in the intestine consist of a real and essential organ known as commensal flora or microbiota. A morphofunctional entity, composed of intestinal microbiota, intestinal epithelium, and mucosal immune system, is responsible for the integrity and homeostasis of gastrointestinal tract. Gut microbial species composition differs greatly among individuals. Each person represents a unique collection of bacterial species, which is highly stable over the time. Variability of gut microbiota is based on the host organism's age, on genetic factors, and on environmental factors [1, 2].

Recent molecular techniques have identified 4 major microbial phyla which represent over 90% of the gut microbiota:* Firmicutes*,* Bacteroides*,* Proteobacteria*, and* Actinobacteria*. The most commensal bacteria present in human fecal flora are represented by two main groups of* Firmicutes*, subdivided in* Clostridium coccoides* (*Clostridium* cluster XIVa) and* Clostridium leptum* (*Clostridium* cluster IV) that are butyrate producers, and by the group of the* Cytophaga-Flavobacterium-Bacteroides* (CFB) [3, 4].

Gut microbiota has metabolic and trophic functions. It has a direct role in the fermentation of dietary residuals and sugar, in the production of substances with antibiotic activity, in the metabolism of proteins, and in the synthesis of vitamins. In addition, it may have a role in the control of proliferation and differentiation of epithelial cells contributing to the formation of a protective barrier against pathogenic organisms [5, 6]. In particular, the fermentation mechanisms of carbohydrates have an important role in the production of short chain fatty acids (SCFA) that are the main source of energy for the enterocytes and are involved in the proliferation and in the differentiation of these cells.

Carbohydrates that arrive in the colon are, in the great part, fibers, and their degradation leads to the production of gas and SCFA such as acetate, propionate, and butyrate. Human body does not possess the majority of hydrolytic enzymes that are involved in these reactions, which are, however, present in the bacterial species forming the gut microbiota [7, 8].

In this review, we will discuss how the intestinal microbiota modifications (intestinal dysbiosis) and the modulation of innate immune response can lead to and exacerbate* Clostridium difficile* infection (CDI).

## 2. Clinical Aspects of* Clostridium difficile* Infection

*Clostridium* (*C.*)* difficile* (*Clostridium* cluster XI) is a Gram-positive anaerobic spore-forming bacillus that lives in the environment (soil, water, and animal feces) and in the human gut where it can be a normal commensal [9]. Indeed, some people are carriers of the bacterium but do not develop the symptoms of the infection. We can refer to CDI only in the presence of symptoms [10, 11]. The disease is caused by toxin A and B expression that is responsible for gastrointestinal illness with a wide spectrum of severity, ranging from mild diarrhea to pseudomembranous colitis, that may progress to toxic megacolon, sepsis, and death [12].

There are several risk factors for* C. difficile*-associated diarrhea (CDAD). In particular, factors like the older age, the presence of comorbidities, an increased exposure to the spores of* C. difficile* during prolonged hospitalizations, and overall protracted and combined antimicrobial therapies can alter gut microbiota and promote CDI [13].

Diagnosis of CDI is based on a combination of clinical presentation signs confirmed by microbiological evidence of* C. difficile* toxin in the stools and, in certain cases, by a lower endoscopic exam that demonstrates pseudomembranous colitis [14].

Current treatment options for CDI are based on the use of oral antibiotics, fecal microbiota transplantation (FMT), or surgery for severe clinical pictures [15]. The antibiotics commonly used to treat CDI are metronidazole, vancomycin, and fidaxomicin. Patients with fulminant CDI who failed to respond to antimicrobial therapies and progress to systemic toxicity with peritonitis and toxic colonic dilatation require surgical intervention such as total colectomy [16]. In recent years, the restoration of healthy gut microbiota by FMT constitutes a suggestive effective therapeutic option for the management of recurrent CDI [17].

## 3. Interaction between Commensal Microbiota and* Clostridium difficile*

A great clinical problem related to CDI is the presence of relapses that are more difficult to treat. In fact, sometimes* C. difficile* may relapse despite a good adherence to the therapy. The meaning of this evidence is not well understood. There are many studies which indicate a role of the microbiota and its alteration in the development of the infection and in the resistance to antibiotic therapy [18, 19]. Intestinal dysbiosis may be due to several mechanisms such as the use of medication, diet, and physical and psychological stress [20] (Tables 1 and 2).

Drugs most frequently implicated in the alteration of the intestinal microbiota are antimicrobic agents. It is proved that the administration of various types of antibiotics, in particular clindamycin, second and third generation cephalosporins, fluoroquinolones, and macrolides, can alter the ratio of different microbial communities. As described in several studies, there is a decrease in carbohydrate-fermenting and butyrate-producing bacteria members of* Bacteroides* and* Firmicutes* phyla [21–25].

A reduction of butyrate producers (such as* Roseburia* and* Ruminococcus*) is observed also in NSAIDs users, particularly in elderly subjects. These subjects, for their natural modification of the gut microbiota related to the age, have already an increased variability of microbial species and a relative decrease of* Firmicutes* and* Bacteroides* regardless of NSAIDs use [26].

Also acid-suppressing agents (H2-receptor antagonists and proton-pump inhibitors) can cause a change in the bacterial flora of the gastrointestinal tract. In particular, there is an increase of gastric and duodenal contamination with a possible minor degradation of* Clostridium* spores by gastric juices [27, 28]. The significance of this observation in the development of CDI is, however, still controversial. In fact, not all researchers recognize a primary role of acid suppression in establishing conditions that favor the* Clostridium* growth [29]. Furthermore, nutrition can have a direct role in modifying the intestinal microbiota and in creating a favorable environment for the growth of* C. difficile*. In particular, a prolonged elemental diet, poor in fibers, which are a substrate for some beneficial bacteria, can support the development of an alteration in the ratio of normal commensal bacteria [24, 30].

Overall, these environmental factors and the consequent intestinal dysbiosis disrupt and alter the protective effect exerted by the gut microbiota against recurrent CDI. The loss of this protective barrier allows for the formation of an ecological niche where* C. difficile* can develop and better resist to antimicrobial therapies.

This niche concept is even more important if we consider that* C. difficile* multiplication and development, facilitated by dysbiosis, are necessary for CDAD [31, 32]. Consequently, intestinal dysbiosis is very important in the pathogenesis of the disease, especially when specific changes in the composition of the gut microbiota occur. CDI patients have a greater diversity of bacterial species and a reduced concentration of some commensal species, in particular the most represented phyla such as* Bacteroides* and* Firmicutes*.* Bacteroides*, which appear to be extremely reduced in these patients, are mainly responsible for the digestion of carbohydrates in the intestinal lumen, resulting in the production of substrates essential for the homeostasis of colonocytes. The reduced concentration of these commensal bacteria has been therefore associated with a higher frequency of relapse of CDI [23, 33, 34].

Also, the components of* Firmicutes* phylum are less represented in CDAD patients with respect to healthy subject. At family level,* Lachnospiraceae *and* Ruminococcaceae*, that are important butyrate producers, are significantly unrepresented in CDI, whereas* Deltaproteobacteria*, that are sulfate-reducing bacteria, are depleted. In contrast, several genera are enriched in association with CDI, such as* Veillonella*,* Enterococcus,* and* Lactobacillus*.

This evident dysbiosis generates an altered production of substrates fermented by the anaerobic gut microbiota, including butyrate, other SCFAs, acetate, and lactate that are critical to the homeostasis of the intestinal epithelial cells [35]. Butyric acid has an important anti-inflammatory molecule and is the preferred source of energy of colonocytes. Other SCFAs are known to decrease intestinal permeability and to increase the production of antimicrobial substances and mucin [36, 37]. Furthermore, a direct role of SCFAs in the inhibition of the growth of* C. difficile *was also assumed. This hypothesis has been confirmed by* in vitro* experiments, but results of* in vivo* studies do not seem to fully confirm this hypothesis [38, 39].

Higher concentration of some species of* Firmicutes* such as* Ruminococcus gnavus*,* Ruminococcus hansenii,* and* Clostridium nexile* was associated with a greater risk of recurrence and development of CDI. These bacterial species are producers of a trypsin-dependent antimicrobial substance (ruminococcin A) that has a low activity against* C. difficile* but can contribute to the disruption of the normal intestinal flora [40]. Another bacterial species that is capable of producing a substance with antimicrobial activity is the* Bacillus thuringiensis*. This bacteria strain produces the Thuricin CD that* in vitro* models proved to inhibit the growth of* C. difficile*. The efficacy of this molecule is effective as well as metronidazole [41, 42].

A further mechanism that gut microbiota uses against the* C. difficile* is the metabolization of bile that is proven to have a role in both the spores germination and the growth of the vegetative form [19]. Commensal flora plays two important roles in bile transformation. A first mechanism is represented by the action of bile salt hydrolase enzymes produced by bacteria. These enzymes transform bile acids by cleaving their glycine and taurine; the metabolites obtained can stimulate the germination of spores. A second mechanism is mediated by the enzyme 7-dehydroxylase that is also produced by the bacterial flora; this enzyme converts primary bile acids, cholate, and chenodeoxycholate into secondary biliar acids: deoxycholic and lithocholic acids, respectively. It is not yet well known which bacterial species operate on the transformation of bile acids [43, 44].

Deoxycholate is a potent germinant but is highly toxic to vegetative cells; cholate stimulates spore germination and vegetative* C. difficile*, whereas chenodeoxycholate has a strong inhibitory effect on spore germination. An alteration in the ratio of the different bile acids, caused by a change in the gut microbiota composition, may promote or inhibit the growth of* C. difficile* [45–47].

In a recent paper, it was demonstrated that the conjugated bile salt taurocholate is able to inhibit* C. difficile* toxins A and B activities in an* in vitro* assay. These results suggest that the mechanism of taurocholate-mediated inhibition modulates toxin activity. Indeed, taurocholate does not appear to affect* C. difficile* growth and toxin production [48].

An additional mechanism that commensal flora uses against the* C. difficile* colonization is represented by the competition for energy sources, in particular carbon source, between toxigenic* Clostridium* and nontoxigenic* Clostridium*. In animal models, it has been shown that nontoxigenic* Clostridium*, prevailing in this competition, crowds out* C. difficile* by ecological niche preventing its growth. Unfortunately, little is still known about this interesting aspect [19, 49, 50].

## 4. *Clostridium difficile* and Innate Immune Response

Several studies on commensal* Clostridia* showed that high levels of metabolite products, and their colonization in close proximity to the intestinal mucosa, are able to exert a strong influence on the host immune system [4]. Indeed, it has been shown that* Clostridia* can promote the development of *αβ* T-cell receptor intraepithelial lymphocytes (IEL) and immunoglobulin A (IgA-) producing cells in the large intestine [51]. IEL, IgA-producing cells within the lamina propria, and intestinal epithelial cells are key players in determining the nature of the immunological response to antigens or pathogens ingested. Umesaki et al. assessed that germ-free mice inoculated with 46 strains of* Clostridia* singly isolated from conventional mice showed an increase in the ratio of CD4^−^ CD8^+^ cells to that of CD4^+^ CD8^−^ in *αβ*IEL within the large intestine. Conversely, the number and phenotype of IEL were similar to those in conventionally housed mice. The number of IgA-producing cells in the colons of mice treated with* Clostridia* was slightly increased compared to that in germ-free mice [51]. Thus,* Clostridia* appear to be involved in the promotion of immunological development [51] in the large intestine, but not in the small intestine. Moreover, commensal* Clostridia* are able to normalize cecal size when they are associated with germ-free mice [52]. How the immune system fundamentally senses* Clostridia* remains unclear. In this context, it has been suggested that the presence or gradient of SCFAs and secondary bile acids produced by* Clostridia* may be sensed by epithelial cells and, in turn, may be associated with the initiation of immunological signaling [51], due to the cross-talk between epithelial and immune cells. For example, IL-7 secreted by epithelial cells can activate IL-7 receptor-bearing IEL on their progenitors [53, 54]. Furthermore, IL-6 [55] and transforming growth factor *β* [56] produced by the epithelia during infection can stimulate the development of Peyer's patches and IgA production [57].

Notably, elevated levels of* Clostridium *clusters* XIVa* and* IV* in mice lead to resistance to allergy and intestinal inflammation in experimental models [58]. Conversely, the microbiota of individuals with chronic inflammation shows lower bacterial diversity and it has been determined that* Clostridium *clusters* IV*, particularly* F. prausnitzii*, and* XIVa* are significantly less abundant in IBD patients compared to healthy subjects [59–61]. It is still unknown whether the decrease in* Clostridia* is a cause or a consequence of chronic inflammation in IBD patients and in autoimmunity, but we can speculate that they are necessary for immune homeostasis, contributing to the suppression of autoimmunity and deleterious inflammation in humans.

### 4.1. Effects of* C. difficile* Toxins Associated with Acute Colitis

In animal models the challenge of ileal loops with* C. difficile* toxin A produces an intense inflammatory response characterized by fluid accumulation, edema, increased mucosal permeability, mast cell degranulation, epithelial cell death, and neutrophil recruitment.

Toxins are able to trigger fluid secretion, to induce the production of reactive oxygen intermediates, IL-8 from colonic epithelial cells [62], and to downregulate mucin exocytosis from mucin-producing colon cells [63].

Moreover, toxins lead to the production of multiple proinflammatory cytokines and chemokines including IL-12, IL-18, interferon g (IFN-g), IL-1b, TNF-a, macrophage inflammatory protein 1 a (MIP-1a), MIP-2, IL-8, and leptin [64]. These factors can exacerbate the inflammation and may be responsible for host damage and many of the histopathological features of* C. difficile*-associated diseases.

Intestinal mast cells also play an important role in the toxin-mediated inflammatory responses. Both toxins A and B lead to activation, degranulation, and the release of inflammatory mediators from mast cells [65]. The inhibition of mast cell degranulation and the blockade of mast cell-derived histamine were associated with a decrease in inflammatory responses to toxin A [66]. Mast cell-deficient mice show severe inflammation and neutrophilic infiltration compared with wild-type mice in response to* C. difficile* toxin A [67]. These studies suggest that, like neutrophils, mast cells propagate the inflammatory response in* C. difficile*-associated diseases. To be noted, a part of the toxin A mediated neutrophil recruitment in rat ileal loops is dependent on mast cell activation [67].

The role of other immune cells, including macrophages, monocytes, and dendritic cells, has generally been extrapolated from* in vitro* and* ex vivo* studies using human and mouse cell lines, human monocytes, and monocyte-derived dendritic cells. Emerging evidence showed also that* C. difficile* toxins can stimulate the release of proinflammatory cytokines and chemokines from macrophages, monocytes, and dendritic cells with a mitogen-activated protein kinase (MAPK-) and p38-dependent pathway [68]. Furthermore, toxin A leads to NF-*κ*B-mediated IL-8 production from human monocytes [69].

### 4.2. Effects on the Innate/Adaptative Immune System Predisposing to Recurrence of CDI

**Figure 1:**
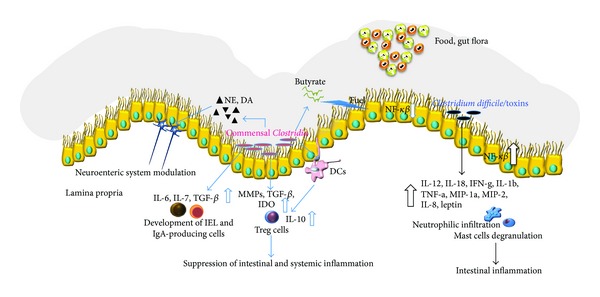
Commensal* Clostridia* have a peculiar role in modulating gut homeostasis. Establishing a close relationship with gut cells (interfold region),* Clostridia* spp. exert a strong influence on the host immune system. On the other hand,* C. difficile* and its toxins lead to the production of multiple proinflammatory cytokines and chemokines including IL-12, IL-18, interferon g (IFN-g), IL-1b, TNF-a, macrophage inflammatory protein 1 a (MIP-1a), MIP-2, IL-8, and leptin [66]. These factors can exacerbate the inflammation and may be responsible for host damage and many of the histopathological features of* C. difficile*-associated diseases.

*C. difficile* is able to modulate intestinal innate immune responses and several groups studied this process.* Clostridium difficile* is able to modulate host innate immunity via toxin-independent and dependent mechanisms [70, 71]. The innate immune mechanisms against the toxins produced by* C. difficile* include the endogenous microbial flora, the mucus barrier, intestinal epithelial cells, and the mucosal immune system. Furthermore,* C. difficile* infection triggers the release of multiple proinflammatory mediators (cytokines, chemokines, and neuroimmune peptides) and the recruitment and activation of several innate immune cells (Figure 1).

Interestingly,* C. difficile* toxins activate both surface and intracellular innate immune sensors, including the inflammasome and the TLR4, TLR5, and NOD1 signaling pathways [72]. TLR4- and MyD88-dependent signaling pathways produce an enhanced inflammatory response [73]. The deficiency of these pathways increases the bacterial burden and the worsening of the disease [73].

*C. difficile* shows a proteinaceous cell surface layer, which is composed of an array of proteins arranged in a crystalline lattice. The surface layer proteins have the ability to activate proinflammatory signaling through TLR4 expressed on the surface of host cells. Engagement of TLR4 initiates downstream signaling of NF-*κ*B and interferon regulatory factor 3, resulting in subsequent production of inflammatory cytokines and immune cell activation. Surface layer proteins induce dendritic cell maturation and activation* in vitro*, as demonstrated by increased expression of major histocompatibility complex class II, CD40, CD80, CD86, and production of IL-12p70, tumor necrosis factor-a, IL-23, and IL-6 [73]. Moreover, surface layer proteins were found to activate NF-*κ*B, but not interferon regulatory factor 3. This indicates that the signaling is myeloid differentiation primary 12 response gene 88 (MyD88)-dependent. In fact, TLR4-deficient and MyD88-deficient mice were more susceptible to infection and exhibited greater pathology than wild-type mice [73]. Increased mucosal damage and inflammation in MyD88-deficient mice were attributed to a lack of neutrophil recruitment to the site of infection [74]. Neutrophils were shown to be critical in preventing bacterial dissemination through damaged mucosa [74]. In the case of TLR5 signaling, exogenous stimulation of TLR5 signaling was protective against* C. difficile* infection [75].

The intracellular innate immune sensors NOD1 and the IL-1b/inflammasome are also activated after* C. difficile* infection [72].* C. difficile*-induced NOD1 activation triggered chemokine production and NOD1-deficient mice have lower chemokine production, less neutrophil recruitment, and more severe disease [72]. In fact NOD1-deficient mice have a higher* C. difficile* burden [72].* C. difficile *toxins stimulate IL-1b release by activating inflammasomes in both mouse macrophages and human colon biopsy specimens [76].

Activation of the innate immune sensors and the release of cytokine and chemokine mediators are followed by an intense local neutrophilic infiltration [77]. This neutrophilic infiltration is one of the major pathological findings after* C. difficile* infection. Local recruitment and systemic proliferation of neutrophils are seen in* C. difficile*-associated diseases [77]. Indeed, induction of neutropenia in rats was associated with less severe disease [78].

## 5. Conclusions

In recent years, several studies analyzed the role of gut microbiota in human physiology and in maintaining gut immune homeostasis. One of the most interesting aspects involves CDI and CDAD.

Intestinal dysbiosis and impaired innate immune response are crucial players in triggering* C. difficile* colonization and related symptoms. In these conditions this Gram-positive anaerobic spore-forming bacillus finds an ecological niche where it can grow and better resist antimicrobial therapies.

In this scenario, gut microbiota modulation and the consequent control of the innate immune response represent a valuable and interesting tool to treat CDI-related diseases.

## Figures and Tables

**Table 1:** This table shows the list of the main factors involved in the development of dysbiosis that promotes recurrent *Clostridium difficile* infection.

Dysbiosis promoting factors	
(i) Antimicrobic agents	
(ii) NSAIDs	
(iii) Acid suppressing agents	
(iv) Age	
(v) Diet	

**Table 2:** This table shows the list of pathogenetic factors generated by dysbiosis.

Pathogenetic factors resulting from dysbiosis	
(i) SCFAs and other fermentative metabolites	
(ii) Bacterial antimicrobic molecule	
(iii) Bile acids metabolism	
(iv) Competition for nutritional sources	
